# New Element Organic Frameworks Based on Sn, Sb, and Bi, with Permanent Porosity and High Catalytic Activity

**DOI:** 10.3390/ma3042447

**Published:** 2010-03-30

**Authors:** Julia Fritsch, Marcus Rose, Philipp Wollmann, Winfried Böhlmann, Stefan Kaskel

**Affiliations:** 1 Department of Inorganic Chemistry, Dresden University of Technology, Mommsenstraße 6, D-01062 Dresden, Germany; E-Mails: julia.fritsch@chemie.tu-dresden.de (J.F.); marcus.rose@chemie.tu-dresden.de (M.R.); philipp.wollmann@chemie.tu-dresden.de (P.W.);; 2 Faculty of Physics and Earth Science, University of Leipzig, Linnéstraße 5, D-04103 Leipzig, Germany; E-Mail: bohlmann@physik.uni-leipzig.de (W.B.)

**Keywords:** element organic frameworks, EOF, heterogeneous catalysis

## Abstract

We present new element organic frameworks based on Sn, Sb and Bi atoms connected *via* organic linkers by element-carbon bonds. The open frameworks are characterized by specific surface areas (BET) of up to 445 m^2^ g^-1^ and a good stability under ambient conditions resulting from a highly hydrophobic inner surface. They show good performance as heterogeneous catalysts in the cyanosylilation of benzaldehyde as a test reaction. Due to their catalytic activity, this class of materials might be able to replace common homogeneous element-organic and often highly toxic catalysts especially in the food industry.

## 1. Introduction

In recent years, the development of new porous materials with potential application in adsorption, separation, gas storage, and heterogeneous catalysis has attracted considerable attention. A rational design was achieved in metal-organic frameworks (MOFs), coordination polymers consisting of metal ions or clusters (connector) and multidentate ligands (linker) defining the network topology and pore diameter [1,2,3,4] and in covalent organic frameworks (COFs) using boronic acids as building blocks [5,6]. They surpass traditional materials such as zeolites and activated carbon in terms of specific surface area (SSA) and pore volume. A disadvantage of these materials is the low hydrothermal stability in some cases [7]. In recent years, several highly porous framework materials have been developed. Due to strong covalent bonds by connection of the organic linkers, materials with a high thermal and hydrolytic stability have been discovered. These metal-free organic frameworks, also known as porous polymers, show interesting properties besides their high stability. For example, the polymers of intrinsic microporosity (PIMs) are soluble and maintain their porosity upon drying [8]. Their pore system is a result of highly rigid chains with a non-efficient space packing resulting in free voids. Since this kind of framework is not cross-linked, PIMs are soluble and maintain their porosity upon drying. In contrast, most of the other known porous polymers consist of a highly crosslinked three-dimensional framework. Thus, they are not soluble. Due to their amorphous structures consisting of slightly flexible organic linkers, the frameworks often show a dynamic behavior upon different states of adsorption. This effect was proven by several groups that published different small angle X-ray scattering (SAXS) curves for one framework material on the one hand under vacuum and on the other hand under a defined pressure at 1 bar [9,10]. The difference in X-ray scattering is a result of a change in the pore size and geometry indicating the flexibility of the framework.

However, it was shown for several metal-free organic frameworks that the modular concept of MOFs can be applied, although the frameworks show no long-range order. A class of materials representing the transition from coordinative cross-linked MOFs to exclusively covalent cross-linked organic framework materials are the element organic frameworks (EOFs). These materials consist of organic linkers that are covalently connected by direct element-carbon bonds. So far, only frameworks connected by silicon atoms were reported. To generate such polymers, an organometallic synthesis route is used. E.g., EOF-2 is obtained by combining the organic linker 4,4’-dibromobiphenyl and tetraethylorthosilicate (TEOS) resulting in a highly stable and porous framework material [11]. Silicon based EOFs are characterized by high hydrophobicity, high stability against water and thermal treatment. Our interest was to generate new EOFs suitable as heterogeneous catalysts. We used precursors based on tin, antimony and bismuth instead of the silicon source TEOS to connect the organic linker molecules to a three-dimensional, porous framework with catalytic active sites. In this work we present three new EOFs based on the organic linker 4,4’-dibromobiphenyl and the elements Sn (EOF-3), Sb (EOF-4), and Bi (EOF-5). Especially the catalytic properties of a heterogeneous tin containing catalyst could be highly interesting for applications in esterification reactions in the food industry. So far, mainly homogeneous tin catalysts are used, showing a high toxicity. A heterogeneous catalyst might be the ideal alternative because it is much easier to separate from the reaction mixture than a homogeneous catalyst. Thus, toxic substances as side products can be avoided.

A method to evaluate the catalytic activity of porous materials is the cyanosilylation of aldehydes or ketones catalyzed by Lewis acids or bases is a convenient test reaction under mild thermal and catalytic conditions. In 1994, Fujita *et al.* already showed the catalytic activity of MOF-based materials using [Cd(4,4’-bpy)_2_](NO_3_)_2_, a two-dimensional square network [12]. Also the catalytic activity of HKUST-1 (Cu_3_(BTC)_2_ with BTC = 1,3,5-benzenetricarboxylate) and MIL-101 were reported [13,14,15]. HKUST-1 is a material characterized by well defined accessible Lewis acid sites, exposed to the inner surface. However, the material shows only moderate yields of 57% (88.5% selectivity) after a duration of 72 h. On the other hand, MIL-101 shows really high catalytic activity in the cyanosilylation of benzaldehyde with product yields of 98.5% after 3 h, even if a very low catalyst loading of only 0.5 mol % is used. A disadvantage of MIL-101 is the chromium content of the material, well known for its toxicity [16]. Besides the synthesis and characterization of structure, composition and adsorption properties of the new Sn-, Sb-, and Bi-containing EOFs, we present the results of the catalytic activity tests in the above mentioned test reaction.

## 2. Results and Discussion

### 2.1. Synthesis and characterization of new element organic frameworks (EOF-3, -4, and -5)

**Scheme 1:**
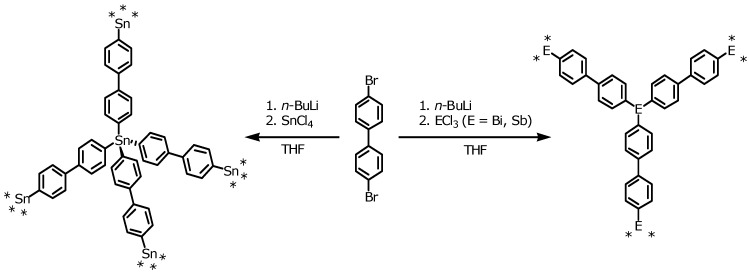
Synthesis of EOF-3 (left), EOF-4, and EOF-5 (right).

The synthesis of three new element organic frameworks based on Sn (EOF-3), Sb (EOF-4), and Bi (EOF-5) atoms as connectors between bifunctional organic linker molecules by direct element-carbon bond is shown in Scheme 1.

With the two-fold lithiation of 4,4’-dibromobiphenyl and subsequent reaction with the respective element chloride, the EOFs can be synthesized in a one-step procedure. The resulting white to pale-grey powders of all three materials are insoluble in common solvents indicating the formation of a three-dimensional highly cross-linked framework. The materials are thermally stable in air up to 473 K (DTA/TG see Figure S1). The stability is a little bit lower than for EOFs containing silicon as connector such as EOF-2 but sufficient for thermal removal of remaining solvent molecules [11].

**Figure 1:**
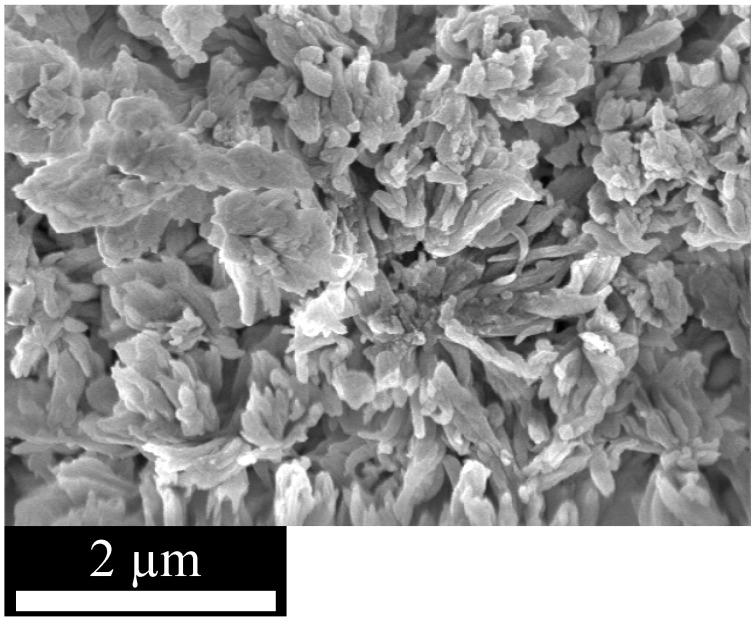
SEM micrograph of EOF-5 shows very heterogeneous particle size and shape distribution. Micrographs of EOF-3 and -4 are not shown but look similar.

The morphology of the particles in the fine powders was investigated using scanning electron microscopy (SEM). The micrographs of all three materials look very similar (Figure 1). They show a very heterogeneous distribution of the particle size in the lower micrometer range and relatively undefined particle shapes.

The framework materials are X-ray amorphous (not shown), as expected due to the kinetic controlled reaction path including the formation of covalent bonds that are irreversible under the present synthesis conditions. The composition, short range order and the close vicinity of linkers and connectors was investigated using elemental analysis (C, H), *Fourier* transformed infrared spectroscopy (FT-IR) and solid state nuclear magnetic resonance spectroscopy (NMR). Under ideal conditions, the products have a chemical composition of Sn(4,4’-biphenyl)_2_, Sb_2_(4,4’-biphenyl)_3_ and Bi_2_(4,4’-biphenyl)_3_ (Scheme 1). The results of the elemental analysis (Table S2) nearly confirm the calculated weight percents of carbon and hydrogen. The differences could be a result of a partial substitution of tin, antimony, or bismuth with aliphatic or hydroxy groups. Further proof is given by FT-IR and solid state NMR analysis described in the following sections.

**Figure 2:**
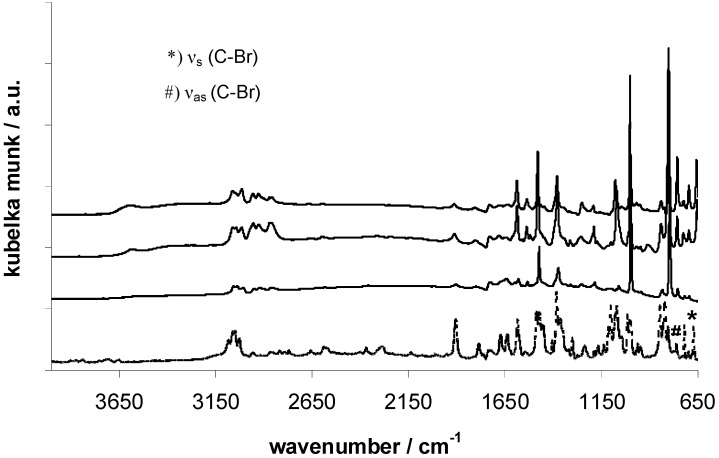
FT-IR spectra of EOF-3 (top), -4 (middle) and -5 (bottom) in comparison with 4,4’-dibrombiphenyl (dashed line).

FT-IR analysis shows the successful halogen-metal-exchange of 4,4’-dibromobiphenyl in all three cases, according to the absence of ν_s_ (C-Br) at 671 cm^-1­^ and ν_as_ (C-Br) at 760 cm^-1^ in the EOF-spectra (Figure 2) [17,18]. The signals at 2950 cm^-1^ indicate an aliphatic substitution of tin, antimony or bismuth as a result of the lithiation with *n*-butyllithium and therefore residual reactive molecules at the moment of the framework formation. Another explanation for the aliphatic groups can be a decomposition reaction of buthyl lithium with the solvent tetrahydrofurane at higher reaction temperatures as mentioned in the literature before [19] and resulting inclusion of aliphatic chains in the framework. But also the aromatic linker molecules show several signals between 3000 and 3100 cm^-1^ as a result of aromatic C-H-group vibrations [20]. The broad band at 3606 cm^-1^ indicates the presence of Sn-OH and Sb-OH groups in the respective EOFs as a result of residual chlorine by reaction with water molecules.

The results of the ^1^H MAS NMR measurement (Figure S2) confirm the presence of aliphatic groups by a signal at a chemical shift of around 0 ppm and the aromatic linkers with a signal maximum at 6.3 ppm. Additionally, the shoulder in the spectrum of EOF-4 at 3.5 ppm indicates the presence of metallic hydroxy groups, confirming the assumption from the FT-IR data presented in Figure 2.

**Figure 3:**
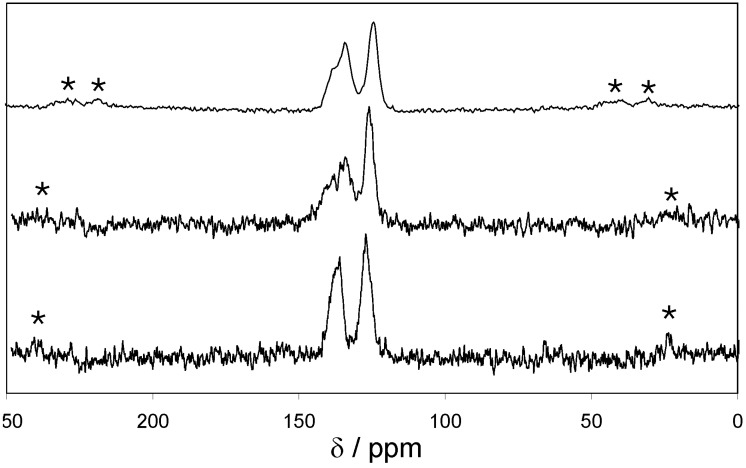
^13^C CP MAS NMR spectra of EOF-3 (top), -4 (middle), and -5 (bottom) (* spinning sidebands).

The ^13^C CP MAS NMR spectra of EOF-3 to -5 (Figure 3) show two strong signals in a chemical shift region of 120–140 ppm resulting from the aromatic biphenyl units as the main compound of the organic frameworks. The ratio of aliphatic groups is too small to be detected by this method. Signals should appear below 40 ppm.

**Figure 4:**
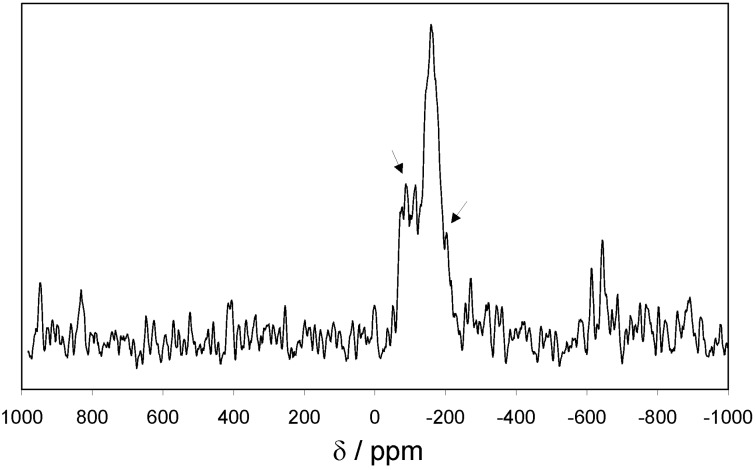
^119^Sn MAS NMR spectra of EOF-3 (→ satellites of ^117^Sn coupling).

Additionally, the tin containing EOF-3 was investigated using ^119^Sn MAS NMR spectroscopy (Figure 4). Only one strong signal at a chemical shift of -160 ppm can be observed, indicating a tin-phenyl-species. All the smaller signals in the close neighbourhood are satellites from the coupling with ^117^Sn. No signal indicating the substitution of tin by aliphatic or hydroxyl groups can be detected.

**Scheme 2:**
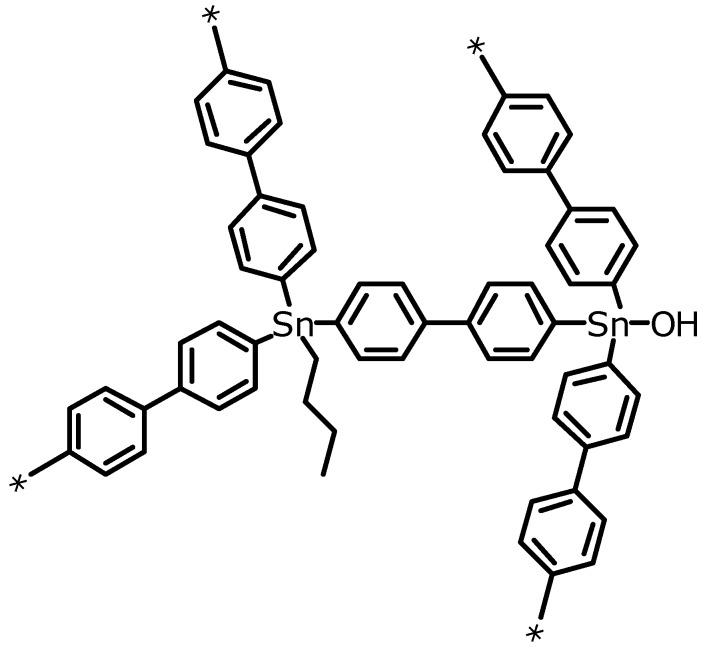
Schematic representation of the three-dimensional framework structure of EOF-3 including additional aliphatic and hydroxy groups.

The main conclusion from the structural investigation of the frameworks by FT-IR and NMR spectroscopy is that the framework consists mainly of aromatic biphenyl units that are connected by the respective elements. Only a small amount of aliphatic substituents from side reactions with the buthyl lithium and maybe from decomposition reactions of buthyl lithium with the solvent tetrahydrofurane can be detected. Also small amounts of hydroxy groups connected to the elements were found (Scheme 2).

### 2.2. Characterization of physisorption properties

**Figure 5:**
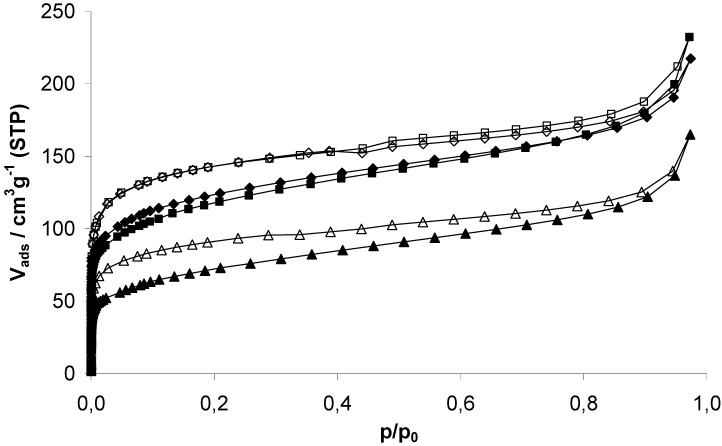
Nitrogen physisorption isotherms of EOF-3 (diamonds), -4 (squares) and -5 (triangles) measured at 77 K.

EOF-3, -4, and -5 show moderate porosity determined by nitrogen physisorption experiments at 77 K (Figure 5). The isotherms shape can be described as a combination of type I and II according to the IUPAC classification. The steep increase in nitrogen uptake at very low pressures indicates the presence of micropores. Further continuous increase of the uptake is a result of the high external surface area due to the presence of very small particles, also indicated by the increasing capacity at p/p_0_ > 0.9 by adsorption in interparticular voids. All isotherms show a hysteresis over the whole pressure range as a result of the amorphous flexible framework. This effect was already described in several publications dealing with porous polymers [9,10].

EOF-3 to -5 exhibit a specific surface area (SSA) of 445, 423 and 261 m^2^g^-1^ (BET), respectively. The t-plot method for determination of the micropore volume and differentiation between internal and external surface area shows a significant ratio of external surface area due to very small particles. All determined values concerning the porosity of the three materials are summarized in Table 1.

The pore size distribution was investigated using the quenched solid density functional theory (QSDFT) equilibrium model for nitrogen on carbon at 77 K with slit pores. So far, there are no models available for materials other than carbon and silica. But for qualitative predictions and the comparison of similar materials other than carbon this method should be sufficient. Especially the organic framework structures should be comparable to porous carbon materials. One should be aware of the general limitations in quantitative determination of pore size distribution.

**Table 1:** Porosity characterization results for EOF-3 to -5.

		EOF-3	EOF-4	EOF-5
SSA_BET_ (p/p_0_ = 0.05-0.15)	m^2^g^-1^	445	423	261
SSA_micro_(t-Plot)	m^2^g^-1^	246	272	127
SSA_external_(t-Plot)	m^2^g^-1^	140	152	134
V_micro_ (*Gurvich*, p/p_0_ = 0.2)	cm^3^g^-1^	0.19	0.18	0.11
V_total_ (*Gurvich*, p/p_0_ = 0.9)	cm^3^g^-1^	0.27	0.27	0.19
V_micro_ (DFT)	cm^3^g^-1^	0.26	0.30	0.22
V_micro_ (t-Plot, p/p_0_ = 0.2-0.5)	cm^3^g^-1^	0.13	0.12	0.05
V_H2O_(p/p_0_ = 0.95)	cm^3^g^-1^	0.044	0.041	0.024
V_H2O_/V_micro_(N_2_,p/p_0_ = 0.2)*100	%	16	15	13
V_ads_(H_2_, STP)	cm^3^g^-1^	60	63	29
H_2_ uptake	wt %	0.55	0.56	0.26

**Figure 6:**
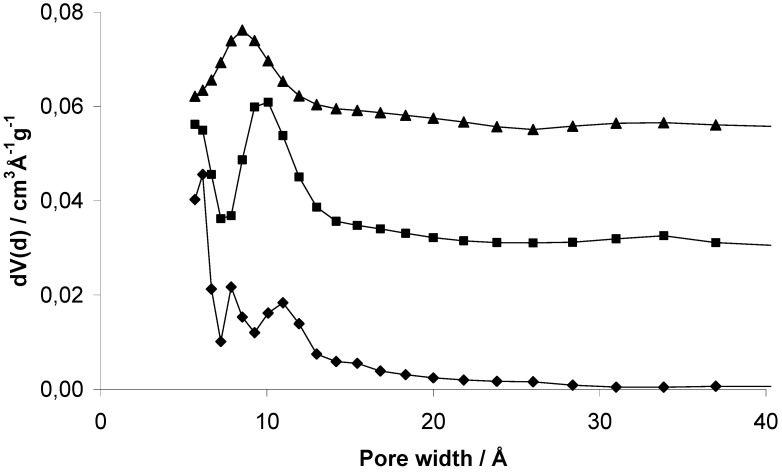
Pore size distribution of EOF-3 (diamonds), -4 (squares, offset: 0.03) and -5 (triangles, offset: 0.055) determined using the quenched solid density functional theory (QSDFT) equilibrium model for nitrogen on carbon at 77 K with slit pores.

However, all three samples show a broad distribution mainly of micropores (Figure 6). EOF-3 shows two maxima at 8 and 11 Å while EOF-4 and -5 exhibit one broad maximum at 10 and 9 Å, respectively. The comparison of the corresponding micropore volumes with different methods (Table 1) leads to very different results. The values determined by DFT are much higher than the values determined by the t-plot method or by the method according to *Gurvich*. The reason for this could be the high ratio of external surface area that is not taken into account using the DFT method.

Hydrogen uptake capacities were measured at 77 K up to a pressure of 1 bar (Figure S3). EOF-3 and -4 show very similar values of 0.55 and 0.56 wt %, respectively. The slightly higher capacity of EOF-4, which has a lower total SSA, can be explained by the higher ratio of internal micropore surface area in comparison to the external surface area determined by the t-plot method (Table 1). As expected, the significantly lower SSA of EOF-5 results in a much lower hydrogen capacity of 0.26 wt %.

**Figure 7:**
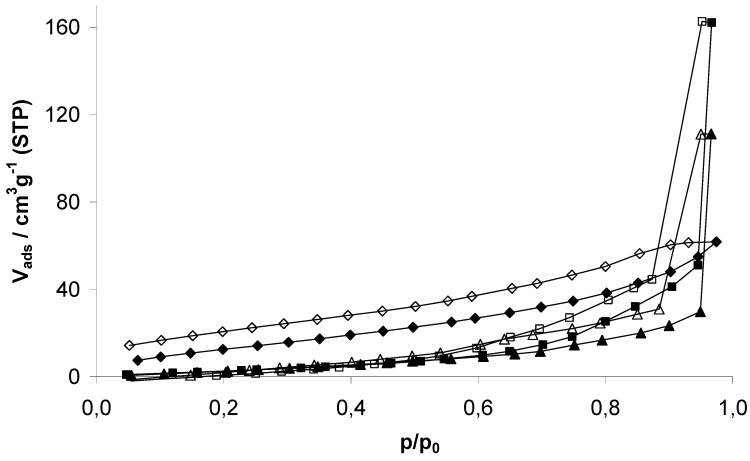
Water vapor physisorption isotherms of EOF-3 (diamonds), -4 (squares) and -5 (triangles) measured at 298 K.

Water vapor physisorption was measured at 298 K to investigate the polarity of the surface (Figure 7). All three materials show very low adsorption at low relative pressure, which is slightly increasing with increasing humidity. At high relative pressure >0.95 a steep increase in water uptake can be observed for EOF-4 and -5, indicating water condensation in the cell maybe due to the high external surface area. The convex shape of the isotherms in relation to the x-axis indicates very weak interactions between the water molecules and the adsorbent surface. A semi-quantitative approach for polarity characterization is the comparison of the pore volumes at defined relative pressures from the water vapor and nitrogen physisorption isotherms. The ratio of these two values can be interpreted as a pore filling degree. Assuming the adsorption begins on more polar sites in the framework, e.g., like Sn-OH groups, water clusters start growing. But the highly non-polar surface on the organic linkers shows too low interaction with water molecules for adsorption. Therefore, remaining voids in the pores that are not filled during water adsorption can be identified. In case of our materials the ratio of the pore volume from water adsorption at p/p_0_ = 0.95 (assumption: all pores are filled and before water condensation in the cell) and from nitrogen adsorption in micropores at p/p_0_ = 0.2 was determined (Table 1). For all three EOFs pore filling degrees between 10-20% were observed, indicating a highly non-polar surface area.

### 2.3. Catalysis

**Scheme 3:**
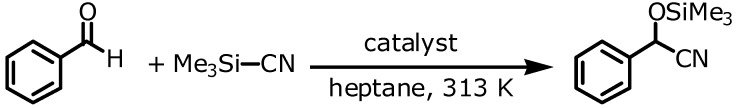
Cyanosilylation of benzaldehyde.

The cyanosilylation of aldehydes or ketones is a convenient route to cyanohydrin derivates using molecular or solid Lewis acid or base catalysts (Scheme 3). Mukaiyama *et al.* found that trimethylsilylcyanide reacts smoothly with aldeydes in the presence of Lewis base catalysts such as tributylphosphine or triphenylantimony in excellent yields [21]. Also the homogeneously catalyzed cyanosilylation of benzaldehyde with triphenylbismuthine was successful [22].

**Figure 8:**
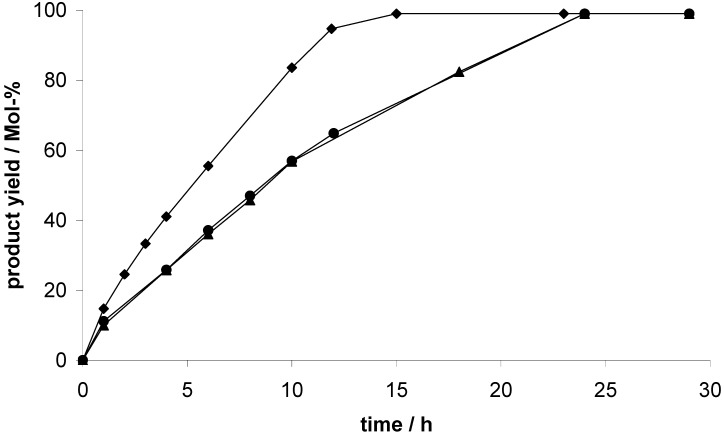
Product concentration during the cyanosilylation of benzaldehyde using EOF-3 (circles), EOF-4 (triangles) and EOF-5 (diamonds) as catalysts.

We used the addition of trimethylsilylcyanide to benzaldehyde as a test reaction to evaluate the catalytic activity of the element organic frameworks (EOF-3 to -5) described in the foregoing sections. Furthermore, the heterogeneity of the reaction mechanism was investigated. For all tests, the respective EOF was activated in vacuum at 353 K overnight. Subsequently, the reactants and the solvent were added and the reaction was monitored using GC-MS analysis. All three materials, EOF-3 as well as EOF-4 and EOF-5, are catalytically active in cyanosilylation of benzaldehyde (Figure 8).

Using EOF-3 and EOF-4 as catalyst, all benzaldehyde is converted after 24 h. With the catalyst EOF-5, a nearly complete consumption is already detected after 12 h. The higher catalytic activity of the bismuth containing EOF-5 in comparison to the antimony analogon EOF-4 confirms the ^1^H MAS NMR data (Figure S2). The shoulder at 3.5 ppm in the spectrum of EOF-4 indicates the presence of metallic hydroxy groups, suggesting a lower number of active centers in EOF-4 compared to EOF-5. 

According to the results presented here, all three EOFs have a higher activity towards the cyanosilylation of benzaldehyde as compared to other porous materials such as the metal-organic framework HKUST-1 showing only moderate yields of 57% after a duration of 72 h [15]. Comparing the catalytic activity with MIL-101 [13], the EOFs show an inferior performance but with regard to the toxicity of chromium the use of MIL-101 is limited.

**Figure 9:**
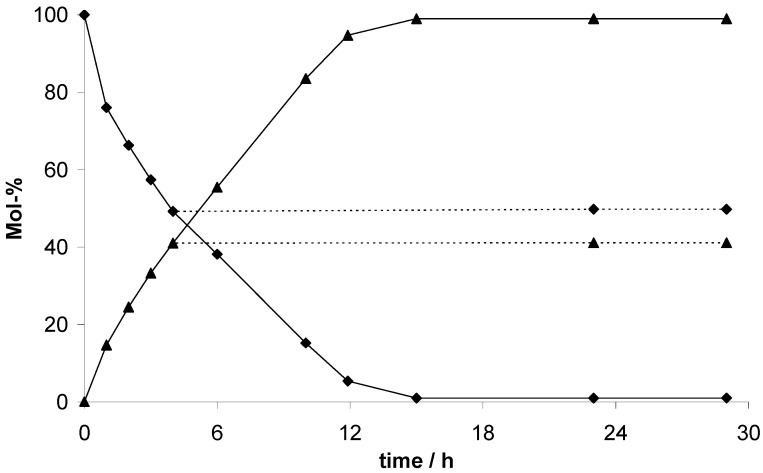
Consumption of benzaldehyde (diamonds) and product yield (triangles) during the EOF-5 catalyzed cyanosilylation with filtration test (dotted lines).

To demonstrate the heterogeneous mechanism of the cyanosilylation catalyzed by the EOFs, the catalyst was filtered off after 4 h (EOF-5) or 6 h (EOF-3 and -4) reaction time and stirring of the filtrate was continued under the same conditions. The reaction mixture was examined with GC-MS analysis again and the concentration of benzaldehyde and the product are shown in Figure 9. The filtration test evidences no change of compound concentration with proceeding reaction time proving the mechanism to be heterogeneous.

**Figure 10:**
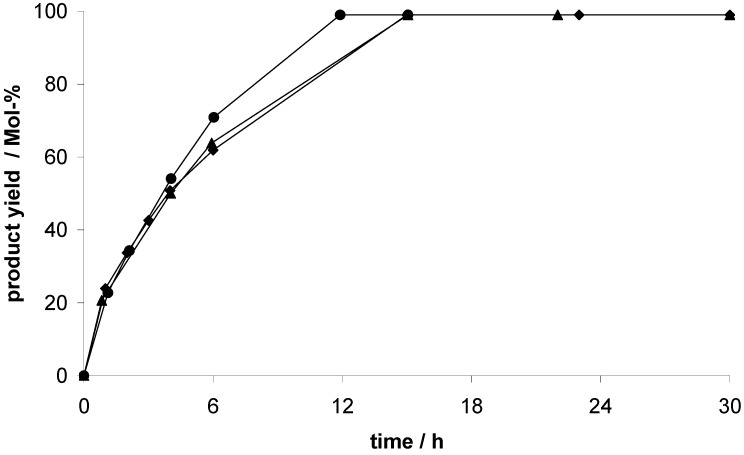
Product concentration during the EOF-5 catalyzed cyanosilylation of benzaldehyde, three consecutive runs were monitored: 1st run (circles), 2nd run (triangles), 3rd run (diamonds).

Considering the recyclability of the catalysts, a recycling test with three consecutive runs was performed. After each run, the catalyst was activated again in vacuum at 353 K overnight. Exemplified for EOF-5, the results of the recycling test are shown in Figure 10. As mentioned before, a product yield of 99% is achieved after 12 h in the first run. In the second and the third run, an almost complete conversion to the product is detected after 15 h. A slight decrease of the reaction rate from the first to the second run is observed but after the second run there is no further loss of activity. Consequently, the EOFs can be reused several times as catalyst in the cyanosilylation of benzaldehyde with faintly reduced activity.

## 3. Experimental Section

### 3.1. General

Commercially available bismuth chloride was sublimed twice. Bismuth- and antimony chloride were handled under argon atmosphere. The tetrahydrofurane for each synthesis was dried using the M. Braun MB SPS-800. Heptane, octane and hexadecane were dried with sodium/benzophenone and stored under argon. Other commercially available chemicals were used as received without further purification (Table S1).

Solid state NMR measurements were carried out at the department of experimental physics at the University of Leipzig. All ^1^H magic angle spinning (MAS) NMR and the ^13^C cross polarization (CP) MAS NMR spectra of EOF-4 and -5 were measured on a Bruker Avance 400 with a resonance frequency of 400.13 MHz for the ^1^H spectra and 100.61 MHz for the ^13^C spectra. All measurements were carried out in a 4 mm ZrO_2_ rotor with a rotation frequency of 10 kHz. For the ^1^H spectra 16 scans with a repetition time of 5 seconds and for the ^13^C spectra 1024 scans with a repetition time of 4 seconds were accumulated. The ^13^C CP MAS NMR spectra were excited with a 4 μs impulse and a mixing time of 3 ms. As external standard tetramethylsilane and adamantane were used. The ^13^C CP MAS NMR spectrum of EOF-3 was measured on a Bruker MSL 500 with a resonance frequency of 125.766 MHz, a rotation frequency of 12 kHz, a 90 degree pulse duration of 5.9 μs and a repetition time of 5 seconds. The CP mixing time was 3 ms and 1000 scans were accumulated. As external standard adamantane with a chemical shift of 37.1 and 28 ppm was used with the Hartmann-Hahn-Matching conditions adjusted. The ^119^Sn MAS spectrum was measured on a Bruker MSL 500 with a resonance frequency of 186.455 MHz, a rotation frequency of 12 kHz, a 90 degree pulse duration of 5.9 μs and a repetition time of 20 seconds. 3800 scans were accumulated. The chemical shift was determined with a solution of SnCl_2_ in water (12 M) with a defined chemical shift of -388.1 ppm.

Infrared (IR) spectra were recorded on a Nicolet Magna 550 Series II between 650 and 4000 cm^-1^ at room temperature, measured in diffuse reflection mode.

Elemental analyses (C, H) were performed with Hekatech EA 3000 Euro Vector CHNSO elemental analyzer at the Elemental Analysis Service, Institute of Organic Chemistry, Dresden University of Technology.

The thermogravimetric analyses were carried out using a Netzsch STA 409 PC Luxx thermal analyzer with heating in air 5 K/min to 973 K.

The nitrogen and hydrogen physisorption isotherms were measured at 77 K using a Quantachrome Autosorb 1-C apparatus. High purity gases were used for the adsorption measurements (nitrogen: 99.999%, hydrogen: 99.999%). Water vapor adsorption was measured at 298 K on the Quanatachrome Hydrosorb 1000. Before all measurements, the samples were outgassed under vacuum at 353 K over night.

To record catalytic data a Shimadzu GCMS QP5000 equipped with a non-polar BPX5 column (5% Phenyl polysilphenylene-siloxane) from SGE was used.

### 3.2. Synthesis of EOF-3 (Sn)

Under argon atmosphere 0.78 g (2.5 mmol) 4, 4’-dibromobiphenyl were dissolved in 50 mL of dry THF. After cooling down the solution to 263 K, 2.0 mL (5.0 mmol, 2.5 M) *n*-butyllithium were added dropwise. The mixture was stirred for 30 minutes and then 0.326 g (1.25 mmol, 0.14 mL) SnCl_4_ were added drop-wise. The mixture was slightly warmed to room temperature and stirring was continued over night. The resulting precipitate was filtered and washed twice with THF, distilled water and ethanol. After drying under vacuum at room temperature the product was obtained as light beige powder. 

### 3.3. Synthesis of EOF-4 (Sb)

Under argon atmosphere 0.78 g (2.5 mmol) 4, 4’-dibromobiphenyl were dissolved in 40 mL of dry THF. After cooling down the solution to 263 K, 2.0 mL (5.0 mmol, 2.5 M) *n*-butyllithium were added drop-wise. The mixture was stirred for 30 minutes and then 0.388 g (1.7 mmol) SbCl_3_ (dissolved in 10 mL of dry THF) were added drop-wise. The mixture was slightly warmed to room temperature and stirring was continued over night. The resulting precipitate was filtered and washed twice with THF, distilled water and ethanol. After drying under vacuum at room temperature the product was obtained as white powder. 

### 3.4. Synthesis of EOF-5 (Bi)

Under argon atmosphere 0.78 g (2.5 mmol) 4, 4’-dibromobiphenyl were dissolved in 40 mL of dry THF. After cooling down the solution to 263 K, 2.0 mL (5.0 mmol, 2.5 M) *n*-butyllithium were added drop-wise. The mixture was stirred for 30 minutes and then 0.536 g (1.7 mmol) BiCl_3_ (dissolved in 10 mL of dry THF) were added drop-wise. The mixture was slightly warmed to room temperature and stirring was continued over night. The resulting precipitate was filtered and washed twice with THF, distilled water and ethanol. After drying under vacuum at room temperature the product was obtained as light beige powder. 

### 3.5. Reaction of trimethylsilylcyanide to benzaldehyde

Before starting the reaction, 50 mg of the respective EOF were activated in vacuum at 353 K over night. After cooling down the flask to 313 K, it was flushed with argon und filled with 17 mL heptane containing octane and hexadecane as internal standards. Subsequently 425 mg (4.0 mmol) freshly distilled benzaldehyde and 794 mg (8.0 mmol) trimethylsilylcyanide were added. The mixture was stirred at 313 K and the reaction was monitored using GC-MS analysis.

For the filtration test, a part of the mixture was filtered off after 240 min (EOF-5) or 360 min (EOF-3 and -4) and transferred into an argon flushed glass flask held at 313 K. Stirring without the catalyst was continued and also monitored using GC-MS analysis.

For the recycling test, the reaction mixture was filtered off after completion of the reaction and the catalyst was activated again in vacuum at 353 K over night. After activation, the catalyst was used a second time for the cyanosilylation of benzaldehyde under the same conditions like the first run. The same procedure was repeated for a third time. The reaction was examined again using GC-MS analysis.

## 4. Conclusions

In summary, we have presented the synthesis of three new element organic frameworks based on the organic linker 4,4’-dibromobiphenyl and connected by the elements tin (EOF-3), antimony (EOF-4), and bismuth (EOF-5). All three products show permanent porosity with specific surface areas of 445, 423 and 261 m^2^g^-1^ (EOF-3 to -5) determined by nitrogen physisorption. A hysteresis over the whole pressure range indicates the flexibility of the amorphous and highly cross-linked polymeric frameworks. The materials are characterized by a highly non-polar surface area determined by water vapor adsorption experiments showing pore filling degrees between 10 and 20%. FT-IR and solid state NMR experiments indicate the presence of hydroxy- and aliphatic groups in the framework of aromatic linkers and the elements tin, antimony and bismuth as connectors. With the cyanosilylation of benzaldehyde as a test reaction we have studied the catalytic properties of the new EOFs. The materials show a good catalytic activity in all three cases. Additionally, the heterogeneity of the reaction mechanism was shown successfully. With regard to other materials like MOFs, the EOFs seem to be a good alternative as materials in heterogeneous catalysis due to their high stability and low toxicity. Especially the tin containing EOF-3 might be a good replacement for often used homogeneous tin catalyst for esterification reactions in the food industry. Upscaling and further catalytic test with regard to defined application fields are work in progress.

## Supporting Information

Supporting information is available online at http://www.mdpi.com/1996-1944/3/4/2447/s1/.

## References

[1] Kitagawa S., Kitaura R., Noro S. (2004). Functional porous coordination polymers. Angew. Chem. Int. Ed..

[2] Yaghi O.M., O'Keeffe M., Ockwig N.W., Chae H.K., Eddaoudi M., Kim J. (2003). Reticular synthesis and the design of new materials. Nature.

[3] Kaskel S. (2002). Handbook of Porous Solids.

[4] Férey G., Mellot-Draznieks C., Serre C., Millange F. (2005). Crystallized frameworks with giant pores: Are there limits to the possible?. Acc. Chem. Res..

[5] Cote A.P., Benin A.I., Ockwig N.W., O'Keeffe M., Matzger A.J., Yaghi O.M. (2005). Porous, crystalline, covalent organic frameworks. Science.

[6] El-Kaderi H.M., Hunt J.R., Mendoza-Cortes J.L., Cote A.P., Taylor R.E., O'Keeffe M., Yaghi O.M. (2007). Designed synthesis of 3D covalent organic frameworks. Science.

[7] Sabo M., Henschel A., Fröde H., Klemm E., Kaskel S. (2007). Solution infiltration of palladium into MOF-5: synthesis, physisorption and catalytic properties. J. Mater. Chem..

[8] McKeown N.B., Budd P.M. (2006). Polymers of intrinsic microporosity (PIMs): organic materials for membrane separations, heterogeneous catalysis and hydrogen storage. Chem. Soc. Rev..

[9] Schmidt J., Weber J., Epping J.D., Antonietti M., Thomas A. (2009). Microporous Conjugated Poly(thienylene arylene) Networks. Adv. Mater..

[10] Weber J., Antonietti M., Thomas A. (2008). Microporous networks of high-performance polymers: Elastic deformations and gas sorption properties. Macromolecules.

[11] Rose M., Böhlmann W., Sabo M., Kaskel S. (2008). Element-organic frameworks with high permanent porosity. Chem. Commun..

[12] Fujita M., Kwon Y.J., Washizu S., Ogura K. (1994). Preparation, Clathration Ability, and Catalysis of a 2-Dimensional Square Network Material Composed of Cadmium(Ii) and 4,4'-Bipyridine. J. Am. Chem. Soc..

[13] Henschel A., Gedrich K., Kraehnert R., Kaskel S. (2008). Catalytic properties of MIL-101. Chem. Commun..

[14] Hwang Y.K., Hong D.Y., Chang J.S., Jhung S. H., Seo Y.K., Kim J., Vimont A., Daturi M., Serre C., Férey G. (2008). Amine grafting on coordinatively unsaturated metal centers of MOFs: Consequences for catalysis and metal encapsulation. Angew. Chem. Int. Ed..

[15] Schlichte K., Kratzke T., Kaskel S. (2004). Improved synthesis, thermal stability and catalytic properties of the metal-organic framework compound CU3(BTC)(2). Microporous Mesoporous Mater..

[16] Goyer R.A. (1996). Toxic Effects of Metals. Casarett and Doull's Toxicology. The Basic science of Poisons.

[17] Barrett R.M., Steele D. (1972). The vibrational spectra and dihedral angles of biphenyl and the 4,4'-dihalogenobiphenyls. J. Mol. Struc..

[18] Pliev T.N., Karpov O.N., Gordienko L.L., Lavrenyuk T.Y. (1974). Infrared and ultraviolet spectra of polyphenyl ether and intermediate compounds of the process. J. Appl. Spec..

[19] Clayden J., Yasin S.A. (2002). Pathways for decomposition of THF by organolithiums: the role of HMPA. New J. Chem..

[20] Hesse M., Meier H., Zeeh B. (2005). Spektroskopische Methoden in der organsichen Chemie.

[21] Kobayashi S., Tsuchiya Y., Mukaiyama T. (1991). A Facile Synthesis of Cyanohydrin Trimethylsilyl Ethers by the Addition-Reaction of Trimethylsilyl Cyanide with Aldehydes under Basic Condition. Chem. Lett..

[22] Wada M., Takahashi T., Domae T., Fukuma T., Miyoshi N., Smith K. (1997). Asymmetric trimethylsilylcyanation of aldehydes utilizing chiral bismuth compounds. A frontier in bismuth mediated synthetic reactions. Tetrahedron Asymmetry.

