# Supplementary material for: New Element Organic Frameworks Based on Sn, Sb, and Bi, with Permanent Porosity and High Catalytic Activity
Source: Materials (Basel). 2010 Mar 30;3(4):2447–62. doi: 10.3390/ma3042447 (PMC5445839; doi:10.3390/ma3042447)

Supporting Information

Table S1. Used chemicals.

Chemical product	formula	purity	company
4,4'-dibromobiphenyl	$C_{12}H_8Br_2$	99%	Acros Organics
n-butyl lithium in hexane	$CH_3(CH_2)_3Li$	2.5 M	Acros Organics
Bismuthchloride	$BiCl_3$	>98%	Acros Organics
Antimonychloride	$SbCl_3$	99+%	Sigma Aldrich
Tinchloride	$SnCl_4$	99% anhydrous	Acros Organics
Benzaldehyde	C_6H_5CHO	≥98%	Acros Organics
Trimethylsilylcyanide	$(CH_3)_3SiCN$	98%	ABCR

Table S2. Results of elemental analysis.

EOF	element	Calculated / wt	1st detection / wt	2nd detection / wt
EOF-3	C	68.13	58.38	58.42
	H	3.81	3.90	3.87
EOF-4	C	61.76	57.87	57.95
	H	3.46	3.74	3.82
EOF-5	C	49.44	44.61	44.59
	H	2.77	2.51	2.52

Figure S1. TG and DTA (broken line) of EOF-3 (left) and -5 (right).

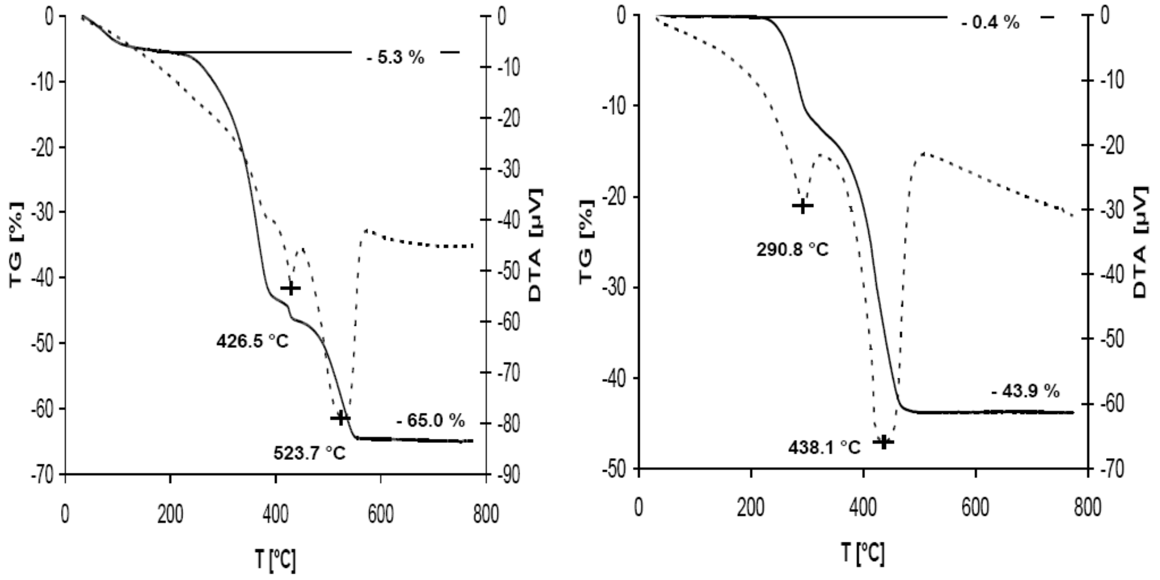

Figure S2. ^{1}H MAS NMR spectra of EOF-4 (top) and EOF-5 (bottom) (* spinning sidebands).

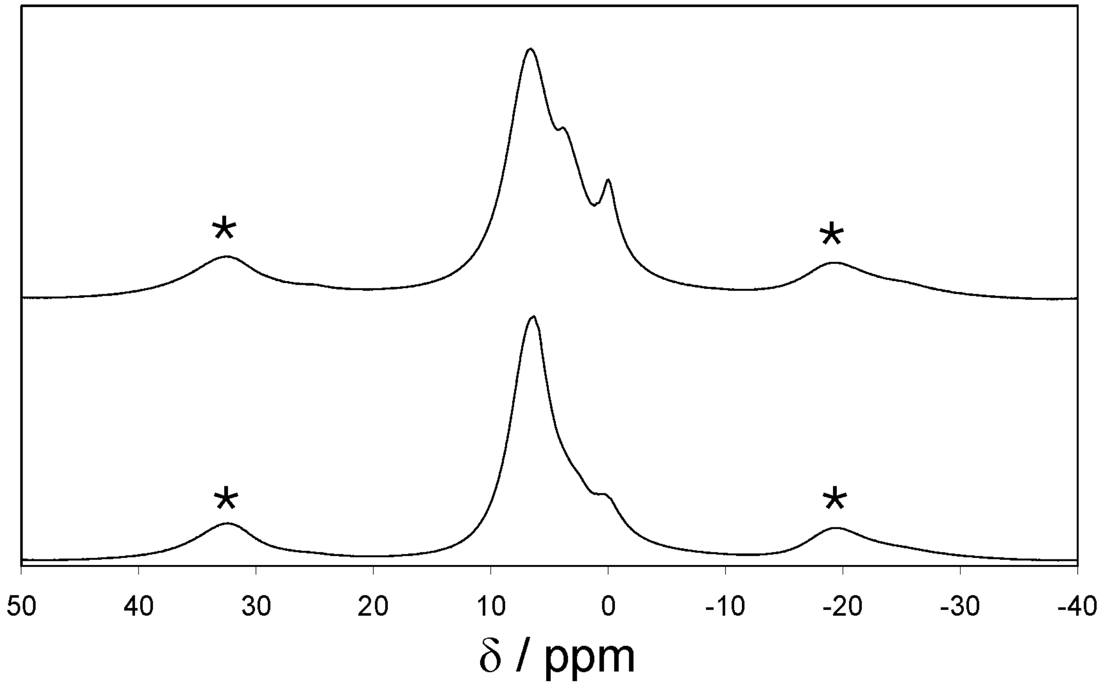

Figure S3. Hydrogen physisorption isotherms of EOF-3 (diamonds), -4 (squares) and -5 (triangles) measured at 77 K.

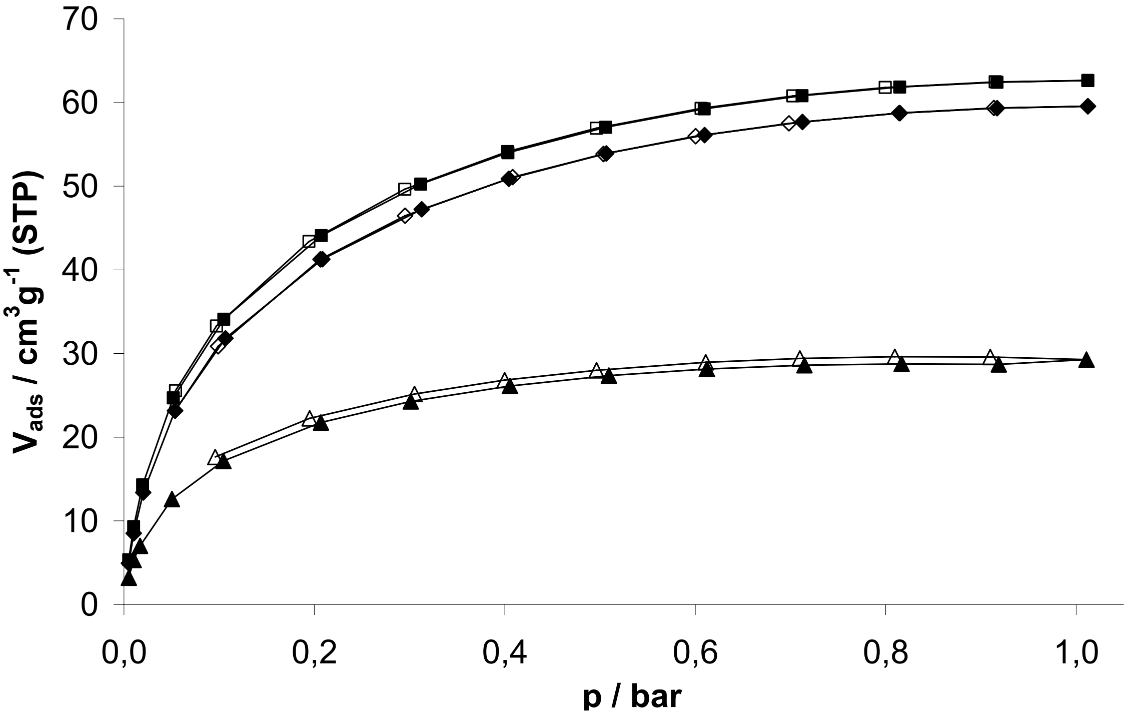

Supplement: Supplementary file 1 [file materials-03-02447-s001.pdf]
